# Treatment outcomes of oral sitafloxacin in acute complicated urinary tract infection and pyelonephritis

**DOI:** 10.1186/s40064-016-2044-5

**Published:** 2016-04-05

**Authors:** Weerawat Manosuthi, Surasak Wiboonchutikul

**Affiliations:** Department of Medicine, Bamrasnaradura Infectious Diseases Institute, Ministry of Public Health, Tiwanon Road, Nonthaburi, 11000 Thailand

**Keywords:** Sitafloxacin, Urinary tract infection, Pyelonephritis, Treatment

## Abstract

**Background:**

Data on the success rate of sitafloxacin treatment in acute complicated urinary tract infection and pyelonephritis are limited.

**Objectives:**

To determine the success rate of a new oral fluoroquinolone, sitafloxacin, in acute complicated urinary tract infection and pyelonephritis.

**Methods:**

A prospective study of 30 patients who were diagnosed with acute complicated urinary tract infection and pyelonephritis was conducted. Oral sitafloxacin 50 mg twice a day was given for 7 days. All patients were followed up at baseline, day 7, and day 14.

**Results:**

The patient sample included 67 % females; the mean ± SD age was 49 ± 13 years. Twenty-one (70 %) patients had acute pyelonephritis and 9 (30 %) had complicated urinary tract infections. Twenty-two patients were positive for uropathogens. The most frequently isolated pathogen was *E. coli* 11 non-extended spectrum beta-lactamase (ESBL) producing and 5 ESBL-producing strains. Of the 22 isolated uropathogens, 19 (86 %) isolates were sensitive to sitafloxacin. At day 14, 29 of 30 (97 %) were clinically cured and 21 of 22 (95 %) were microbiologically cured. No patients discontinued sitafloxacin due to adverse events.

**Conclusions:**

These results support the use of oral sitafloxacin in complicated urinary tract infections and acute pyelonephritis. However, further larger studies are required to confirm these results.

## Impacts of findings on practice statements

- Oral sitafloxacin demonstrated high rates of microbiological success in the treatment of acute complicated UTI and pyelonephritis.
- Oral sitafloxacin demonstrated high rates of clinical success in the treatment of acute complicated UTI and pyelonephritis.
- Overall tolerability profile of sitafloxacin is favorable.

## Background

Complicated urinary tract infections and acute pyelonephritis are relatively common infectious genitourinary problems. A complicated urinary tract infection localizes to the lower or upper urinary tract and is related to structural or functional abnormalities that may increase the risk of treatment failure, urinary tract obstruction and urinary bladder dysfunction (Grabe et al. 2013). Acute pyelonephritis is commonly described as an infection of the upper urinary tract that includes fever and flank pain. It is an exudative purulent localized inflammation of the renal pelvis and kidney (Grabe et al. 2013). These are potentially serious infections that require prompt and effective antibiotic therapy. Renally excreted fluoroquinolones, such as levofloxacin and ciprofloxacin, are frequently prescribed for treatment (Hooton 2003). For patients who can tolerate oral therapy, empiric treatment with an oral fluoroquinolone is an optimal treatment. Fluoroquinolones used to previously treat urinary tract infections include ciprofloxacin, levofloxacin, norfloxacin and ofloxacin (Gupta et al. 2011; Singh et al. 2013). A previous study of 28 hospitals in Thailand showed that 44 % of *Escherichia coli* isolated from the urine was resistant to ciprofloxacin and found a correlation between the incidence of ciprofloxacin-resistant *E. coli* bacteremia and the upward trend in quinolone use in the community (Polwichai et al. 2009). In 2008, sitafloxacin, a potent broad-spectrum oral fluoroquinolone, was launched (Keating 2011). This drug is mainly renally excreted as an unchanged drug (Payne et al. 2005; O’Grady et al. 2001). Sitafloxacin possesses activity against a variety of uropathogens, including *E. coli* (Keating 2011). Based on susceptibility rates, the activity of sitafloxacin against *E. coli* appeared greater than that of levofloxacin or ciprofloxacin. The bioavailability of sitafloxacin is 100 %, and food intake does not affect the pharmacokinetics of this drug to a significant extent (Nakashima et al. 1995). Approximately 80 % of sitafloxacin was excreted in the urine within 72 h of administration (Keating 2011). Oral sitafloxacin 50 mg twice daily was considered an effective dosage in patients with complicated urinary tract infections (Keating 2011). For the patients suspected of having a poor response to the usual dose, the dosage may be increased to 100 mg twice daily and extended to a 14-day period. To date, data regarding the success rate of sitafloxacin in acute complicated urinary tract infection and pyelonephritis are relatively limited. In addition, the causative uropathogens may vary in each geographic area. Thus, the objective of this study was to examine the treatment’s success rate in Thai patients diagnosed with acute complicated urinary tract infection and pyelonephritis.

## Methods

The present study was designed as a pilot prospective single-arm study involving 30 patients at the Bamrasnaradura Infectious Diseases Institute, Ministry of Public Health, Nonthaburi, Thailand. This trial was performed in compliance with the Declaration of Helsinki and approved by the institutional review boards. Written informed consent was obtained from every patient before the initiation of the study procedures. The period of enrolment was from November 2013 to October 2014. The primary objective was to study the rate of microbiological eradication in patients at day 14. The secondary objectives were to study microbiological eradication at day 7, clinical success at day 14, and the tolerability to sitafloxacin. Inclusion criteria were as follows: (1) patients who were 18–70 years of age, (2) those with complicated urinary tract infections or acute pyelonephritis, and (3) those who participated and provided informed consent. A complicated urinary tract infection was defined as a urinary infection occurring in a patient with a structural or functional abnormality of the genitourinary tract, urinary tract stone, urinary tract obstruction, neurogenic bladder, or indwelling urethral catheter, for example. Exclusion criteria were as follows: (1) patients who could not tolerate oral therapy, (2) those having significant immune deficiency, such as receiving chemotherapy or having human immune deficiency virus infection, (3) having septic shock or unstable vital signs, (4) having prostatitis, perinephric abscess, or intra-renal abscess, (5) history of allergy to fluoroquinolones, (6) underlying diabetes mellitus, (7) history of epilepsy, and (8) myasthenia gravis. Prostatitis was excluded by a historical and physical examination, including digital rectal examination and ultrasonography. One dose of systemic intravenous antibiotic treatment was allowed within 24 h prior to enrolment, including a third-generation cephalosporin or aminoglycoside.

All eligible patients received oral sitafloxacin 50 mg twice a day for 7 days. The treatment period was extended up to 14 days, depending on the treatment response, including the delayed resolution of fever and/or symptoms for more than 5 days. The dosage was increased from 50 mg twice a day to 100 mg twice a day in cases of clinical improvement, despite the sensitivity indicating sitafloxacin resistance. The patients had follow-up visits at day 7 and day 14 after initiation of sitafloxacin, at which time they were assessed clinically and blood samples were taken. Patients provided midstream urine samples for examination; these were sent for culture before the first dose of study medication and subsequent visits. Urine samples were obtained by straight catheterization if urine catheters were placed. Levels of >10^5^ CFU/mL uropathogens in midstream urine in women, >10^4^ CFU/mL in midstream urine in men, or in straight catheter urine in women, were considered to be significant uropathogens in complicated urinary tract infections. A level of >10^4^ CFU/mL uropathogens in midstream urine was considered to be significant in acute uncomplicated pyelonephritis in women. In vitro susceptibility tests of sitafloxacin were performed by the disk diffusion method. *Escherichia coli* ATCC25922, *Pseudomonas aeruginosa* ATCC 27853, and *Enterococcus faecalis* ATCC29212 were used as reference strains. Inhibition zone diameters ≥19 mm and ≤15 mm were the breakpoints for susceptibility for sensitive and resistant gram-negative uropathogens and gram-positive uropathogens isolated from urine. An inhibition zone diameter >15 to <19 mm was an intermediate breakpoint for these uropathogens.

Laboratory parameters, including complete blood cell count, kidney enzymes, erythrocyte sedimentation rate (ESR), C-reactive protein (CRP), and liver enzymes were assessed at the baseline visit and on day 7. The dosage of sitafloxacin was 50 mg per day for patients with estimated glomerular filtration rate (eGFR) ≥30 to <50 mL/min/1.73 m^2^ and 50 mg every other day for patients with eGFR 10 to <30 mL/min/1.73 m^2^. The eGFR was calculated by the Modification in Diet in Renal Disease (MDRD). The eGFR MDRD was calculated as the following: 186× serum creatinine^−1.154^ × age^−0.203^ × (0.742 if female). Subjects whose initial uropathogens were resistant to sitafloxacin could remain in the study if they improved clinically; in this case, they were included in the analysis. In such cases, the dosage of sitafloxacin was adjusted to 100 mg twice daily. Clinical cure was assessed by the investigator and defined as resolution of initial clinical signs and symptoms without antibiotic treatment. Microbiological response was assessed according to results of the urine culture at follow-up visits. “Microbiological cure” was assigned to patients whose urine culture demonstrated elimination of their study entry uropathogens. At day 14, patients were assigned a response of “presumed microbiological cure” if a urine culture was not available at day 14 but a microbiological cure at day 7 combined with a clinical cure at day 14 was documented. Adverse events included events reported by patients and those observed as laboratory abnormalities.

The analyses were performed using the intention-to-treat principle. Frequencies and the mean ± SD were used to describe clinical variables and laboratory variables. The paired *t* test was used to compare means of variables between the baseline visit and day 7. A *P* value <0.05 was considered statistically significant. All analyses were performed using SPSS version 15.0 (SPSS Inc., Chicago, IL, USA).

## Results

Table 1 shows the baseline characteristics of 30 patients. Of the total, 67 % were female; the mean ± SD age was 49 ± 13 years. Sixty percent of patients were treated with outpatient antibiotic therapy and 40 % received inpatient therapy. Twenty-one (70 %) patients had acute pyelonephritis, and 9 (30 %) had complicated urinary tract infections. Twenty-two patients had positive uropathogens. The most frequently isolated pathogens included 11 non-extended spectrum beta-lactamase (ESBL)-producing *E. coli* and 5 ESBL-producing *E. coli*. Of 22 isolated uropathogens, 19 (86 %) isolates were sensitive to sitafloxacin. Two sitafloxacin-resistant isolates included *Enterobacter cloacae* and *Klebsiella pneumoniae* and one sitafloxacin-intermediate isolate was methicillin-resistant *Staphylococcus aureus* (MRSA). Among 16 isolates with *E. coli*, including 11 non- ESBL producing strains and 5 ESBL-producing strains, all were sensitive to sitafloxacin. All 5 ESBL-producing strains were resistant to ciprofloxacin, norfloxacin, cefotaxime, ceftazidime, and cefepime. Of 7 strains resisted to norfloxacin and ciprofloxacin, 5 strains were sensitive to sitafloxacin 2 strains resisted to sitafloxacin, including *E. cloacae* and *K. pneumoniae*.

**Table 1:** Baseline characteristics of 30 patients

Parameters	Number (%)
*Patients’ parameters*	
Sex	
Male	10 (33 %)
Female	20 (67 %)
Age, years, mean ± SD	49 ± 13
Body weight, kg, mean ± SD	62 ± 12
*Laboratory parameters*	
Hematocrit, mg %, mean ± SD	37 ± 6
White blood cells count, cells/mm^3^, mean ± SD	11,340 ± 5,496
Platelet, cells/mm^3^, mean ± SD	305,433 ± 115,971
Neutrophil, %, mean ± SD	75 ± 14
ESR, millimeters per hour, mean ± SD	46 ± 29
C-reactive protein, mg/L, mean ± SD	35 ± 31
BUN, mg/dl, mean ± SD	15 ± 11
Creatinine, mg/dl, mean ± SD	1.13 ± 0.76
Aspatate aminotransferase, U/L, mean ± SD	33 ± 34
Alanine aminotransferase, U/L, mean ± SD	37 ± 45
Total bilirubin, mg/dl, mean ± SD	1.4 ± 3.9
Albumin, mg/dl, mean ± SD	4.0 ± 0.5
Urinary tract infection (UTI) diagnosis	
Acute pyelonephritis	21 (70 %)
Complicated UTI	9 (30 %)
Stroke with neurogenic bladder	3
Catheter-related UTI	2
Ureteric stone with hydronephrosis	2
UTI in men	2
Positive urine culture	22 (73 %)
*E. coli*	11
ESBL producing *E. coli*	4
ESBL producing *E. coli* + ESBL producing *K. pneumoniae*	1
*Enterobacter cloacae*	1
*Enterococcus fecalis*	1
*Klebseilla pneumoniae*	1
*Morganella morganii*	1
MRSA	1
Coagulase negative Staphylococcus	1
Sensitivity to sitafloxacin, n = 22	
Sensitive	19 (86 %)
Intermediate	1 (5 %)
Resistant	2 (9 %)

**Fig. 1:**
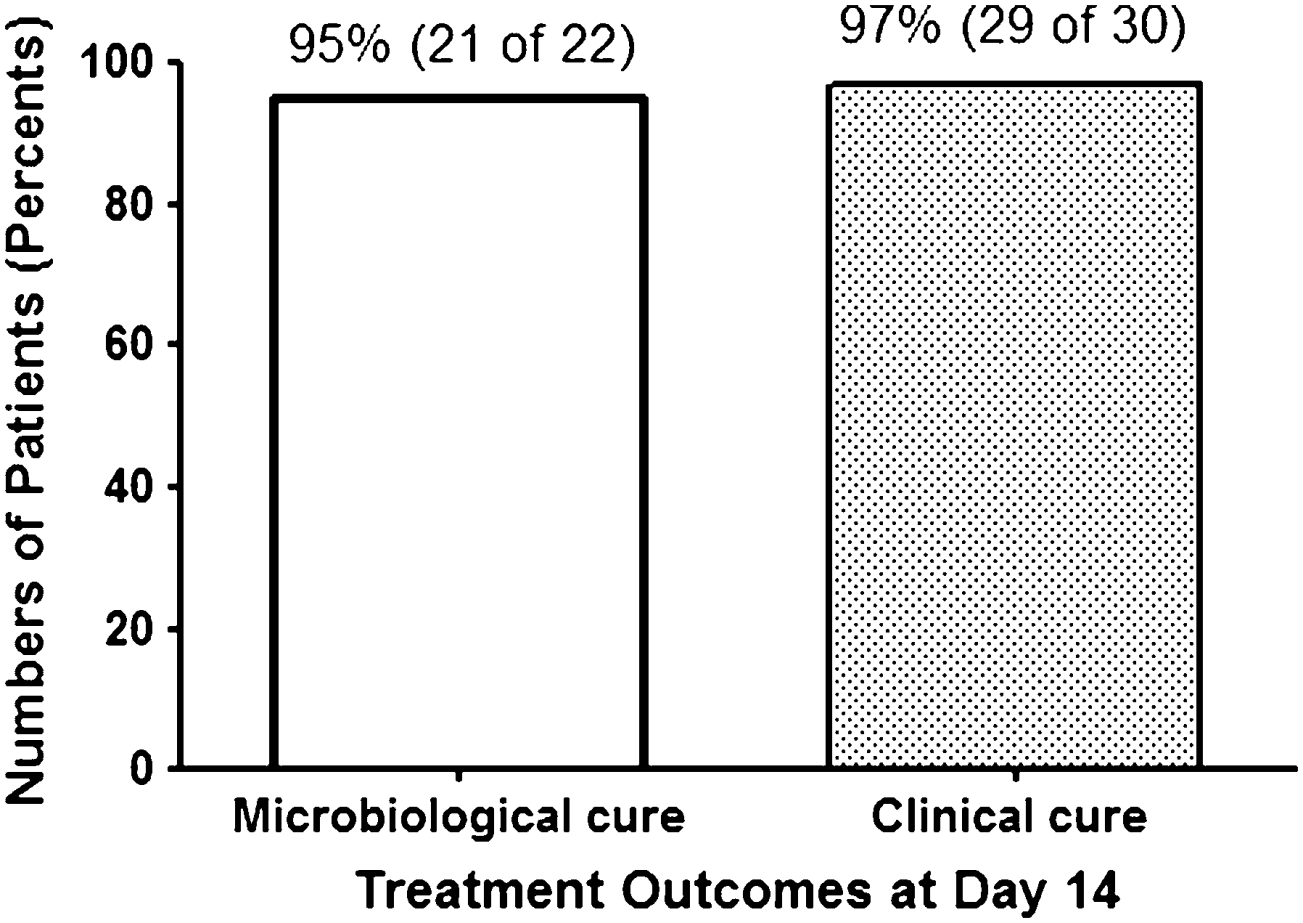
Treatment outcomes at day 14 after sitafloxacin therapy

In terms of treatment response at day 7, among the 22 positive urine cultures at baseline visits, 21 (95 %) were microbiologically cured. Two of 21 patients were presumed to have a microbiological cure. The mean ± SD white blood cell count in the complete blood count was 7710 ± 3452 cell/mm^3^ and the mean ± SD percentage of neutrophils was 58 ± 11 %. The ESR was 50 ± 30 mm per hour. The C-reactive protein was 13 ± 18 mg/L. By repeated measurement analysis, there was a significant decline in C-reactive protein from the baseline visit (*P* < 0.001) but not in ESR (*P* = 0.505). At day 14, 29 of 30 (97 %) patients were clinically cured and 21 of 22 (95 %) patients were microbiological cured as shown in Fig. 1. One female patient with clinical failure was diagnosed to have acute pyelonephritis with ESBL-producing *E. coli*. This isolate was resistant to ciprofloxacin and norfloxacin but not sitafloxacin. Her clinical symptoms worsened after 5 days of treatment, so sitafloxacin was switched to intravenous ertapenem. In terms of tolerability, none of the patients discontinued sitafloxacin due to adverse events. At day 7, the mean ± SD aspartate aminotransferase was 22 ± 7 U/L and the mean ± SD alanine aminotransferase was 30 ± 17 U/L. By repeated measurement analysis, there were no differences in aspartate aminotransferase (*P* = 0.105) and alanine aminotransferase (*P* = 0.390) between the baseline visit and day 7. No initially isolated uropathogens developed resistance to sitafloxacin during treatment.

## Discussion

In the treatment of complicated urinary tract infections and acute pyelonephritis, determining the underlying disease and prompt and effective antibiotic treatment are essential. *E. coli* was the most commonly isolated pathogen and few gram-positive uropathogens were isolated in the present study. Of note, one-third of them were ESBL-producing strains. In addition, a previous report in Thais demonstrated that the incidence of ESBL-producing *E. coli* septicemia was 9.9 cases per 10,000 hospital admissions. The risk factors associated with ESBL-producing *E. coli* septicemia were hospital acquisition, previous use of a fluoroquinolone, and the use of a central venous catheter (Anunnatsiri et al. 2012). Our findings highlight a concern that there was a high rate of drug-resistant uropathogens among the causative uropathogens and a broad range of uropathogens in this setting. This may be partly explained by the fact that this study involved patients with complicated urinary tract infections. In general, drug-resistant uropathogens are more commonly isolated, and the causative uropathogens vary widely when compared to isolates from patients with uncomplicated urinary tract infections. Nonetheless, almost all patients’ infections were microbiologically eradicated.

Sitafloxacin demonstrated high rates of microbiological and clinical success in this study, i.e., >90 %. Kawada and colleagues demonstrated that >90 % clinical efficacy was achieved with sitafloxacin 50 mg twice daily for 7 days (Kawada et al. 2008a). In addition, a previous study demonstrated that >80 % of isolates from patients with complicated urinary tract infections remained susceptible to sitafloxacin, while the rates for levofloxacin and third-generation cephalosporins were lower (Kawada et al. 2008b). Fluoroquinolone resistance in *E. coli* mainly develops by mutations of DNA gyrase and topoisomerase IV (Hoshino et al. 1994). The recommended sitafloxacin dosage for treatment of acute pyelonephritis and complicated urinary tract infections was 50–100 mg twice daily for 7–14 days. The inhibitory activity of sitafloxacin against such enzymes was greater than that of fluoroquinolones (Okumura et al. 2008; Onodera et al. 1999). The MIC90 of sitafloxacin, levofloxacin, ciprofloxacin, and moxifloxacin against *E. coli* clinical isolates were 1–2, 16, 16–32, and 16–32 μg/mL, respectively (Keating 2011). For fluoroquinolone, the maximum concentration (Cmax): minimum inhibitory concentration (MIC) ratio and area under the curve (AUC):MIC ratio are appropriate parameters to predict activity and likelihood of developing drug resistance (Keating 2011). A previous study demonstrated that sitafloxacin 50 mg oral twice daily achieving the Cmax:MIC ratio >5 were associated with eradication rate of 96 % in respiratory tract pathogen. (Kohno et al. 2013) The dosage of sitafloxacin 100 mg oral twice daily achieved a higher Cmax. In addition, mutation prevention concentration (MPC) of sitafloxacin had a lower concentration level than other quinolones. This suggests that sitafloxacin has a higher barrier to developing resistance and the majority of levofloxacin-resistant *E. coli* remains susceptible to sitafloxacin. Two sitafloxacin-resistant isolates were found in the present study without treatment failure. In addition, a double dose of sitafloxacin was allowed to treat these patients to overcome this effect. Previous studies showed that sitafloxacin was more active than levofloxacin, ciprofloxacin and moxifloxacin against bacteria isolated from Thai and Japanese patients including ESBL-producing gram-negative strains (Tiengrim et al. 2012; Amano et al. 2013; Nakamura et al. 2014; Matsumoto et al. 2012). Nevertheless, drug-resistance is one of the major challenges in treating these urinary tract infections due to an increased incidence of fluoroquinolone-resistant uropathogens (Czaja et al. 2007).

The most frequently reported adverse reactions of sitafloxacin included diarrhea and transaminitis (Matsumoto et al. 2012). Diarrhea symptoms were usually mild, as in previous reports (Hooton 2003; Matsumoto et al. 2012). None of the patients discontinued sitafloxacin due to adverse events. Likewise, no elevation of aspartate aminotransferase or alanine aminotransferase was found (*P* > 0.05). Therefore, the overall tolerability profile of sitafloxacin is favorable. A limitation of the present study is that the sample size was relatively small and there were no comparators. Additional larger studies and longer follow-up periods are needed to confirm our findings. In addition, the minimum inhibitory concentration (MIC) of sitafloxacin was not addressed. However, the correlation of the inhibition zone diameter of sitafloxacin as determined by the disk diffusion method and the MIC determined by the agar dilution method has been demonstrated (Thamlikitkul and Tiengrim 2014).

Oral sitafloxacin 50–100 mg twice daily administered for 7–14 days was effective in the treatment of complicated urinary tract infections and acute pyelonephritis. All patients tolerated sitafloxacin well. This result supports the use of oral sitafloxacin against such infections. However, further larger studies are required to confirm these results.
